# Thyroid cancer cell lines: an overview

**DOI:** 10.3389/fendo.2012.00133

**Published:** 2012-11-16

**Authors:** Manuel Saiselet, Sébastien Floor, Maxime Tarabichi, Geneviève Dom, Aline Hébrant, Wilma C. G. van Staveren, Carine Maenhaut

**Affiliations:** ^1^School of Medicine, IRIBHM, Université Libre de BruxellesBrussels, Belgium; ^2^Welbio - Université Libre de BruxellesBrussels, Belgium

**Keywords:** cell line, thyroid, cancer, mutation, WRO, FTC133, TPC1, BCPAP

## Abstract

Human thyroid cancer cell lines are the most used models for thyroid cancer studies. They must be used with detailed knowledge of their characteristics. These *in vitro* cell lines originate from differentiated and dedifferentiated *in vivo* human thyroid tumors. However, it has been shown that mRNA expression profiles of these cell lines were closer to dedifferentiated *in vivo* thyroid tumors (anaplastic thyroid carcinoma, ATC) than to differentiated ones. Here an overview of the knowledge of these models was made. The mutational status of six human thyroid cancer cell lines (WRO, FTC133, BCPAP, TPC1, K1, and 8505C) was in line with previously reported findings for 10 genes frequently mutated in thyroid cancer. However, the presence of a BRAF mutation (T1799A: V600E) in WRO questions the use of this cell line as a model for follicular thyroid carcinoma (FTC). Next, to investigate the biological meaning of the modulated mRNAs in these cells, a pathway analysis on previously obtained mRNA profiles was performed on five cell lines. In five cell lines, the MHC class II pathway was down-regulated and in four of them, ribosome biosynthesis and translation pathways were up-regulated. mRNA expression profiles of the cell lines were also compared to those of the different types of thyroid cancers. Three datasets originating from different microarray platforms and derived from distinct laboratories were used. This meta-analysis showed a significant higher correlation between the profiles of the thyroid cancer cell lines and ATC, than to differentiated thyroid tumors (i.e., PTC or FTC) specifically for DNA replication. This already observed higher correlation was obtained here with an increased number of *in vivo* tumors and using different platforms. In summary, this would suggest that some papillary thyroid carcinoma or follicular thyroid carcinoma (PTC or FTC) cell lines (i.e., TPC-1) might have partially lost their original DNA synthesis/replication regulation mechanisms during their *in vitro* cell adaptation/evolution.

## Introduction

The experimental study of human cancers uses *in vitro* and *in vivo* models. Among the various possible experimental models, human cancer cell lines are frequently used (van Staveren et al., 2009). They have retained hallmarks of cancer cells; they are pure, genetically identical, easily propagated and can be genetically manipulated. A cell line originates from a tissue and is obtained by selection of the most rapidly proliferating and resistant cells in monolayer during passages.

Results obtained on a cell line are sometimes directly extrapolated for *in vivo* cancers which produced this cell line (Yeung et al., 2007; Wang et al., 2008). However, the representativity of the cancer cell line may be distorted by a cross contamination of one cell line by another (Ribeiro et al., 2008; Schweppe et al., 2008), an *in vitro* evolution of the cell line (van Staveren et al., 2009), a strong genomic instability due to the number of passages or a risk of infection (Harlin and Gajewski, 2008). Thus, a systematic verification of the status of these cell lines is important (van Staveren et al., 2007; Ribeiro et al., 2008; Schweppe et al., 2008).

Thyroid cancer is the most frequent endocrine cancer (Kondo et al., 2006; Sipos and Mazzaferri, 2010). There are various types of thyroid carcinomas, the carcinomas from thyrocyte are largely the most frequent: papillary thyroid carcinoma (PTC), follicular thyroid carcinoma (FTC) anaplastic thyroid carcinoma (ATC) and an intermediate form between PTC/FTC and ATC, the poorly differentiated thyroid cancer. Each type is characterized by a set of mutations leading to increased cellular proliferation and dedifferentiation (Catalano et al., 2010).

PTC is the most frequent type of human thyroid carcinoma (Kondo et al., 2006). The genetic alterations most often found in PTC are *BRAF* point mutations, accounting for 40–60% of the cases, and RET/PTC rearrangements which are present in about 20% of the cases. The most frequent *BRAF* mutation occurs in the serine/threonine kinase domain (V600E) and leads to the constitutive kinase activity of the protein (Xing, 2010). This mutation could be a key mutation for the treatment and the diagnosis of the most aggressive PTC (Nucera et al., 2010; Xing, 2010). RET/PTC rearrangements are the result of a fusion between the 3′ end of a receptor of the tyrosine kinase family (RET) and the 5′ end of a gene constitutively expressed in thyrocytes. The most frequent rearrangements of this type are RET/PTC1 and RET/PTC3. The fusion results in the constitutive activation of the truncated tyrosine kinase portion of RET by autophosphorylation due to the dimerization domain of the heterologous gene (Catalano et al., 2010).

The genetic changes most often found in FTC are *RAS* point mutations (approximately 45% of the cases) and PAX8/PPARγ rearrangements (approximately 35% of the cases). Mutations of the *RAS* gene activate the mitogenic MAPK and the PI3K pathways. PAX8/PPARγ rearrangements are the result of a fusion between the 5′ end of the *PAX8* transcription factor and the 3′ end of *PPAR*γ, a member of the nuclear hormone receptor superfamily, constitutively expressed in thyrocytes. Different rearrangements between the 2 genes have been described, and sometimes reported within a single tumor (Lacroix et al., 2004). PAX8/PPARγ chimeric proteins have a dominant negative effect on the wild-type PPARγ. In addition to these, alterations in the tumor suppressor gene *PTEN* (10% of the cases) and the *PI3KCA* oncogenes (10% of the cases) have been described in this tumor type.

ATC is the most dedifferentiated, aggressive thyroid cancer (Kondo et al., 2006). The genetic alterations most often observed are the ones already described in PTC and FTC with the exception of RET/PTC and PAX8/PPARγ rearrangements, but in addition also include mutations in *TP53* and β-*catenin* genes.

For each type of human thyroid carcinoma, derived cell lines have been generated. In this study, we used six different human cancer cell lines derived from different types of thyroid cancers. The WRO (Estour et al., 1989) and FTC133 (Goretzki et al., 1990) cell lines are derived from FTC; the BCPAP (Fabien et al., 1994) commonly known as a PTC cell line is derived from a poorly differentiated PTC; TPC1 (Tanaka et al., 1987) and K1 (Challeton et al., 1997) cell lines are derived, respectively, from PTC and from a metastasis of a well-differentiated PTC (Ribeiro et al., 2008); the 8505C (Ito et al., 1993) cell line from ATC. These six cell lines are among those mostly used for thyroid cancer research. The mutational status of each of these cell lines was investigated and compared to the literature. Next, pathways were further investigated by using our previously obtained data on mRNA expression patterns of these cell lines that had been compared to primary cultured normal human thyrocytes (van Staveren et al., 2007). Previously we showed that mRNA expression profiles from different human thyroid tumor cell lines, including cell lines of the present study, evolved *in vitro* into similar phenotypes with mRNA expression profiles closer to undifferentiated *in vivo* thyroid tumors (ATC) than to differentiated thyroid cancers (PTC and FTC) (van Staveren et al., 2007). To investigate this further, a correlation analysis between mRNA expression profiles from cell lines and mRNA expression profiles from each *in vivo* thyroid tumor type was performed by using three datasets generated in two different laboratories that were derived from different microarray platforms.

## Materials and methods

### Cell culture

Cell lines were obtained from various laboratories. The WRO, FTC133, BCPAP, and 8505C cell lines were received from Prof. G. Brabant (Medizinische Hochschule, Hannover, Germany); the TPC1 cell line from Prof. M. Mareel (University of Ghent, Belgium); the K1 cell line from Dr. Zaruhi Poghosyan (Cardiff University, Scholl of Medecine, Cardiff, UK). The BCPAP, TPC1 and 8505C cell lines were cultured in RPMI 1640 (w L-glutamate) completed by foetal bovine serum (10%), penicillin/streptomycin (2%) and amphotericine B (1%); the FTC133, WRO and K1 cell lines were cultured in Dulbecco's Modified Eagle Medium, Nutrient mixture F-12 (1:1, by volume) completed by foetal bovine serum (10%), penicillin/streptomycin (2%), amphotericine B (1%). All culture reagents were purchased from Gibco (Paisely, UK).

### RNA extraction

After removal of the culture medium, 2 ml of TRIzol Reagent (Ambion, Austin, USA) was added to the cells grown in 10 cm dishes. Total RNA was extracted according to the manufacturer's instructions, followed by purification on miRNeasy columns (QIAGEN, Hilden, Germany). RNA was spectrophotometrically quantified, and its integrity was verified by automated gel electrophoresis (Experion, Bio-Rad, Hercules, USA).

### RT-PCR

After a DNase treatment with DNase I Amplification Grade kit (Invitrogen, Carlsbad, USA), 1 μg of total RNA was used for reverse transcription using hexamers [(3.6 μg/μl) (Roche, Basel, Switzerland)] and reverse transcriptase (Superscript II RNase H Reverse Transcriptase kit, Invitrogen). The PCR reactions were performed with the recombining Taq DNA polymerase kit (Invitrogen). Each PCR reaction was performed in the presence of 5 μl 10X PCR buffer, 1.5 μl MgCl_2_ (50 mM), 1 μl dNTP mix (10 mM, Invitrogen), 1 μl of the forward and the reverse primer (10 μM each), 0.4 μl Taq DNA polymerase (5U/μl), 2 μl DNA from the RT reaction and 38.1 μl of water. Primers and PCR conditions are detailed in Table 1.

**Table 1:** **Targeted exons, sequences of the primers used for PCR amplification, amplicon length and PCR conditions for each investigated gene**.

**Gene**	**Targeted exons(s)**	**Forward primer (5′ –> 3′)**	**Reverse primer (5′ –> 3′)**	**Size (bp)**	**PCR conditions**
BRAF	14 and 15	GCACAGGGCATGGATTACTT	GATGACTTCTGGTGCCATCC	194	Std but Tm at 55°C
NRAS	3	CGCACTGACAATCCAGCTAA	TCGCTTAATCTGCTCCCTGT	255	Std
HRAS	3	GGAAGCAGGTGGTCATTGAT	ACGTCATCCGAGTCCTTCAC	204	Std
KRAS	3	AGAGAGGCCTGCTGAAAATG	TTGACCTGCTGTGTCGAGAA	200	Std
TP53-1	2, 3, 4, 5	GTGACACGCTTCCCTGGAT	ACACGCAAATTTCCTTCCAC	658	Std
TP53-2	5, 6, 7	CCCTTCCCAGAAAACCTACC	AGCTGTTCCGTCCCAGTAGA	518	Std
TP53-3	6, 7, 8, 9, 10, 11	GCTGCTCAGATAGCGATGGT	GTGGGAGGCTGTCAGTGG	660	Std
PI3KCA-1	9, 10, 11	TGACTGGTTCAGCAGTGTGG	GGCCAATCTTTTACCAAGCA	341	Std
PI3KCA-2	20, 21	TTTTGACACAGGATTTCTTAATAGTGA	GGTCTTTGCCTGCTGAGAGT	418	Std but Tm at 55°C
PAX8/PPARγ	Pax8: 8, 9, 10 PPARγ: 3 8, 9 8, 10 8, 9	GCAACCTCTCGACTCACCAG (PAX8)	CATTACGGAGAGATCCACGG (PPARγ)	407 305 217 108	Std
RET/PTC1	RET:12, 13 H4: 1	GGCACTGCAGGAGGAGAAC (H4)	GATGACATGTGGGTGGTTGA (RET)	277	Std
RET/PTC3	RET:12, 13 ELE1: 7	AAGCAAACCTGCCAGTGG (ELE1)	TGCTTCAGGACGTTGAAC (RET)	240	Std but with 30 cycles
PTEN-1	4, 5, 6	GACATTATGACACCGCCAAA	CGCCACTGAACATTGGAATA	405	Std
PTEN-2	6, 7, 8, 9	GCTACCTGTTAAAGAATCATCTGGA	TGACGGCTCCTCTACTGTTTT	530	Std but Tm at 48°C
PBGD	11, 12, and 13	AAGGACCAGGACATCTTGGA	AACTGTGGGTCATCCTCAGG	266	Std but Tm at 55°C

Std: Standard conditions: 94°C for 5 min followed by 35 cycles consisting of incubations at 94°C for 30 s, 60°C (Tm) for 1 min and 72°C for 1 min. At the end of cycles the PCR mixtures were incubated at 72°C for 10 min. PBGD gene was used as a positive control for amplification. For each PCR, a negative control (the amplification mix without DNA) was used to detect possible contamination.

### Sequencing

PCR products were purified with the QIAquick PCR purification kit (QIAGEN) according to the manufacturer's instructions. Sequencing was performed with the BigDye Terminator V3.1 Cycle Sequencing Kit (Applied Biosystems, Foster City, USA) with the sequencer ABI PRISM 3130 (Applied Biosystems) and the genetic analysis program 3130-XI. Sequences were analyzed with BLAST (Basic Local Alignment Search Tool program) from NCBI (http://blast.ncbi.nlm.nih.gov/Blast.cgi).

### Pre-processing of the different datasets

A merged file was created which contained three different datasets derived from three different microarray platforms. The first dataset consisted of five cell lines hybridized on IRIBHM custom slides, using primary cultured normal thyrocytes as a reference (www.ulb.ac.be//medecine/iribhm/microarray/data/) (van Staveren et al., 2007). The second dataset was composed of 11 ATC, 49 PTC, and 45 normal thyroid tissues hybridized on the Affymetrix HG U133 Plus 2.0 platform (GSE33630). The third dataset contained 4 ATC, 51 PTC, 13 FTC, and 4 normal thyroid tissues hybridized on the Affymetrix HG U133A platform (Giordano et al., 2005) (GSE27155). Each platform has been validated in previous studies [Giordano et al., 2005; van Staveren et al., 2006; Hébrant et al., 2012; Dom et al. (submitted)]. For both independent Affymetrix platforms, mRNA expression profiles of normal tissues were pooled as a reference and an mRNA expression ratio between each tumor and the normal reference was calculated for each gene. For the comparison of cell lines with *in vivo* thyroid tumors, only genes were included that was expressed in both cell lines and primary thyrocytes. Finally, the merged file contained the mRNA expressions of 2392 genes, expressed in log_2_, present on the three different microarray platforms.

### DAVID pathway analysis

In order to investigate the biological meaning of the modulated mRNAs, Database for Annotation, Visualization and Integrated Discovery (DAVID pathway analysis) (Huang da et al., 2009) was used. For the cell line analyses, only genes showing a regulation of at least |0.58 | in log_2_, i.e., 1.5-fold or more were included. To calculate the background, all the genes present on the IRIBHM custom slides were used. For the comparison between ATC and cell lines, only significant commonly regulated genes were considered. The background was calculated using all the genes present in the merged file. For both analyses, a pathway was selected when its False Discovery Rate (FDR) was <5%. The analysis was performed separately for up-regulated and down-regulated genes.

### Correlation of mRNA expression between *in vitro* human thyroid cancer cell lines and *in vivo* human thyroid tumors

Spearman's correlation coefficients were computed using R (version 2.14.1). Based on the merged file, correlation coefficients between gene expression profiles of ATC and thyroid cancer cell lines were compared to correlation coefficients between gene expression profiles of PTC or FTC and thyroid cancer cell lines, using a Mann–Whitney *U*-test. This assessed whether ATC and cell lines had more similar mRNA expression profiles than FTC or PTC and cell lines. Comparisons of those coefficients were made between samples hybridized on the same microarray platform. 2D-multidimensional scaling was plotted based on those Spearman correlation coefficients using the R function isoMDS from the package MASS v.7.3–18.

### Analysis of the genes commonly and oppositely modulated in ATC *in vivo* and cancer cell lines

Scores and *q*-values were computed based on a slightly modified version of Rank Products (Breitling et al., 2004) to find genes regulated in ATC compared to normal tissues and in cell lines compared to primary cultured normal thyrocytes. Rank Products is a non-parametric method allowing to study differential expression and to integrate the information of different datasets regardless of the potential technical differences, e.g., from different laboratories or different microarray platforms. It has been shown to outperform SAM (Zang et al., 2007) and is more powerful with fewer samples than *t*-test based statistics. The genes with a *q*−value ≤ 0.05 were selected from ATC and cell lines. Genes that were regulated in the same direction in ATC and cell lines were kept and are shown in a heatmap using R library: gplots v.2.10.1 (http://cran.r-project.org/web/packages/gplots/index.html). Similarly, genes that had significant regulations in opposite directions between ATC and cell lines were further analyzed.

## Results

### Mutational status

**Figure 1:**
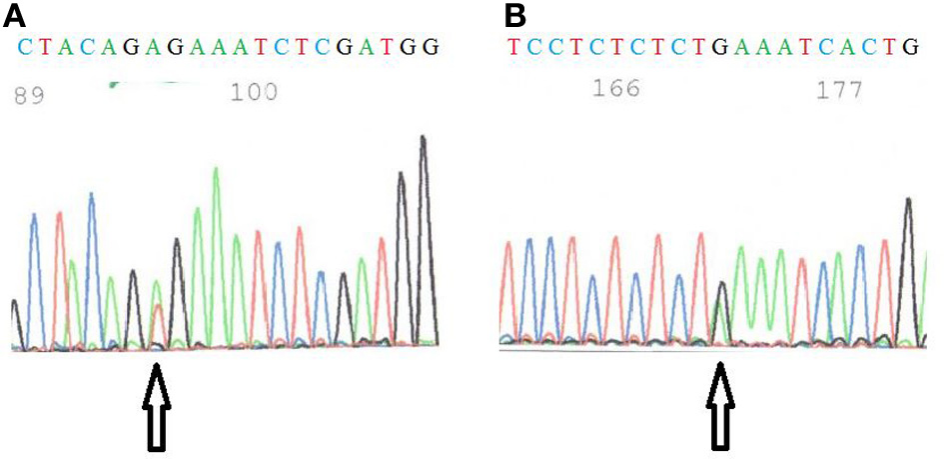
**(A)** Sequence of *BRAF* in WRO cells. The arrow shows the T1799A mutation. **(B)** Sequence of *PI3KCA* in K1 cells. The arrow indicates the G1624A mutation.

The mutational status was investigated for known thyroid oncogenes and tumor suppressor genes in six human thyroid cancer cell lines. For each cell line, the presence of point mutations in *BRAF, NRAS, HRAS, KRAS, TP53, PI3KCA, PTEN* genes was explored as well as the presence of RET/PTC1, RET/PTC3, and PAX8/PPARγ rearrangements. Therefore, primers were designed to target the point mutations and the rearrangements most frequently found in thyroid carcinomas (Kondo et al., 2006) and each mutation was verified by sequencing both DNA strands. The results are summarized in Table 2. Neither *NRAS, HRAS, KRAS* point mutations nor RET/PTC3 or PAX8/PPARγ gene rearrangements were detected in any of the six cell lines. As reported earlier, FTC133 cells showed *PTEN* and *TP53* mutations, BCPAP and 8505C cells showed *BRAF* and *TP53* mutations, and TPC1 cells harbored the RET/PTC1 gene rearrangement. WRO cells presented the heterozygous *BRAF* T1799A (V600E) mutation and K1 the heterozygous *PIK3CA* G1624A (E542K) mutation, in addition to the *BRAF* mutation (Figure 1).

**Table 2:** **Mutational status of six human thyroid cancer cell lines derived from three types of thyroid carcinomas**.

**Cell lines**	**BRAF**	**NRAS**	**HRAS**	**KRAS**	**PI3KCA**	**PTEN**	**TP53**	**RET/PTC1**	**RET/PTC3**	**PAX8/PPARγ**
**WRO (FTC)**	GTG-> GAG V600E^*^	wt	wt	wt	wt	wt	wt	−	−	−
**FTC133 (FTC)**	wt	wt	wt	wt	wt	CGA->TGA R130STOP	CGT->CAT R273H	−	−	−
**B-CPAP (PTC)**	GTG-> GAG V600E	wt	wt	wt	wt	wt	GAC->TAC D259Y	−	−	−
**TPC1 (PTC)**	wt	wt	wt	wt	wt	wt	wt	+	−	−
**K1 (PTC)**	GTG-> GAG V600E^*^	wt	wt	wt	GAA->AAA E542K^*^	wt	CGA->CGC R213R	−	−	−
**8505c (ATC)**	GTG-> GAG V600E	wt	wt	wt	wt	wt	CGG->GGG R248G	−	−	−

Differences between these results and the published data are indicated in bold. These results were compared to previously published data for WRO (Namba et al., 2003; Xu et al., 2003; Garcia-Rostan et al., 2005; Palona et al., 2006; Schweppe et al., 2008), FTC133 (Ricarte-Filho et al., 2009), B-CPAP (Garcia-Rostan et al., 2005; Meireles et al., 2007; Ricarte-Filho et al., 2009), TPC1 (Meireles et al., 2007; Ricarte-Filho et al., 2009), K1 (Garcia-Rostan et al., 2005; Meireles et al., 2007; Ricarte-Filho et al., 2009) and 8505c (Garcia-Rostan et al., 2005; Meireles et al., 2007; Ricarte-Filho et al., 2009; Nucera et al., 2010) cell lines.

* , heterozygous mutation; wt, wild-type genotype (no mutation); +, presence of the gene rearrangement; −, absence of the gene rearrangement.

### Pathway analysis of the cell lines

To define the modulated pathways in thyroid cancer cell lines, the genes from previously published mRNA expression profiles from five thyroid cancer cell lines (FTC133, BCPAP, 8505C, TPC1, WRO) were further analysed (van Staveren et al., 2007). These data reported the differential mRNA expression between each cell line and a pool of human primary cultured normal thyrocytes. DAVID pathway analyses were performed on each individual cell line (Supplementary information A). Four of the five cell lines, i.e., WRO, BCPAP, TPC1, and 8505C, showed commonly regulated pathways. An upregulation of ribosome biosynthesis and translation was detected in these four cell lines, and in addition, expression of genes involved in the cell cycle was increased in WRO and BCPAP cells. Expression of genes involved in DNA replication was up-regulated in both BCPAP and 8505C cells. Furthermore, in all cell lines, expression of genes involved in MHC class II biosynthesis was down-regulated and in four cell lines (not in 8505C) the expression of genes involved in negative regulation of cell death/apoptosis was also down-regulated. In both 8505C and TPC1 cells, expression of genes involved in the immune response was decreased and for the latter, a decrease in the protease inhibitor pathway was also observed. In contrast, the FTC133 cell line showed some differences in the regulated pathways. Although the downregulation of the MHC class II and the negative regulation of cell death/apoptosis were present, no increase of expression of genes involved in ribosome biosynthesis and translation were observed. Instead, FTC133 cells showed a strong upregulation of global RNA processing.

### Correlations between mRNA expression profiles of *in vitro* thyroid cancer cell lines and mRNA expression profiles of *in vivo* thyroid tumors

**Figure 2:**
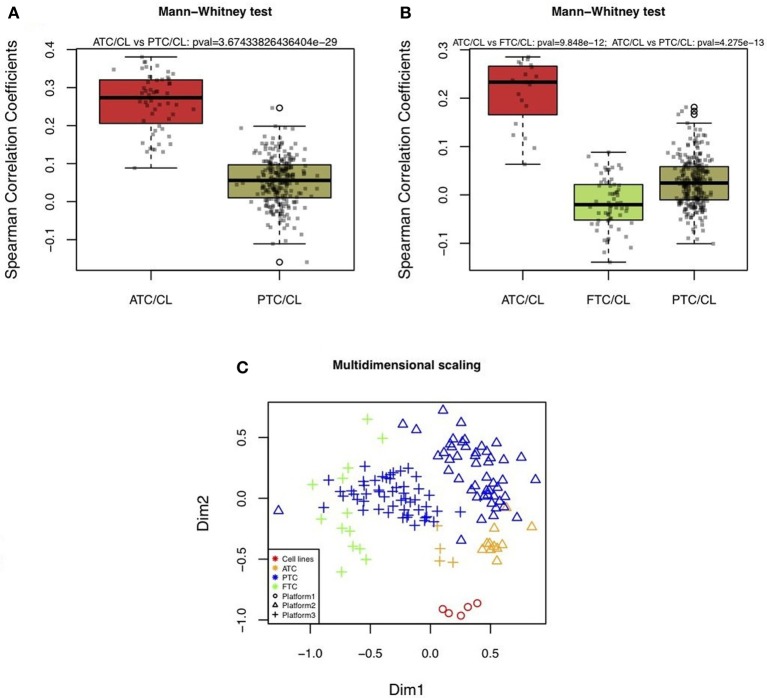
**Boxplots of correlations between cell lines and *in vivo* thyroid tumors**. (**A** and **B**) Correlations were calculated based on 2,392 mRNA expressions comparing human thyroid cancer cell lines (CL) with different types of *in vivo* human thyroid tumors by using three datasets. The first was composed of five thyroid cancer cell lines (WRO, FTC133, BCPAP, TPC1, 8505C) hybridized on IRIBHM custom slides. The second was composed of 11 ATC and 49 PTC hybridized on Affymetrix HG U133 Plus 2.0 **(A)**. The third was composed of 4 ATC, 13 FTC, and 51 PTC hybridized on Affymetrix HG U133A **(B)**. *P*-values indicate whether ATC and cell lines had more similar profiles than FTC or PTC and cell lines. *P*-values were significant for both independent datasets. **(C)** 2D-multidimensional scaling representation of the mRNA expression profiles of cell lines, ATC, PTC, and FTC based on 2392 mRNA expressions.

Two different datasets of mRNA expression profiles from *in vivo* human thyroid tumors were correlated to the mRNA profiles of the WRO, FTC133, BCPAP, TPC1, and 8505C cell lines. One dataset was composed of 11 ATC and 49 PTC hybridized on Affymetrix HG U133 Plus 2.0 arrays. The other dataset consisted of 4 ATC, 13 FTC, and 51 PTC hybridized on Affymetrix HG U133A arrays. For both independent datasets, the Spearman correlation coefficient was the highest between cell lines and ATC compared to the cell lines and PTC or FTC (Figures 2A,B). Furthermore, there was a significant difference between the correlations of ATC versus cell lines compared to those calculated with the PTC or FTC profiles (Figures 2A,B).

These results are in line with previous results which showed by 2D-multidimensional scaling analysis the highest similarity between cell lines and ATC, compared to cell lines with PTC or FTC (van Staveren et al., 2007). Thus, this already observed relation could be confirmed in the present study using more tumor samples that had been hybridized on different microarray platforms. As shown in Figure 2C, although a platform effect was visible, in both independent datasets ATC were closer to cell lines.

### Analysis of the genes commonly and oppositely modulated in ATC *in vivo* and cancer cell lines

**Figure 3:**
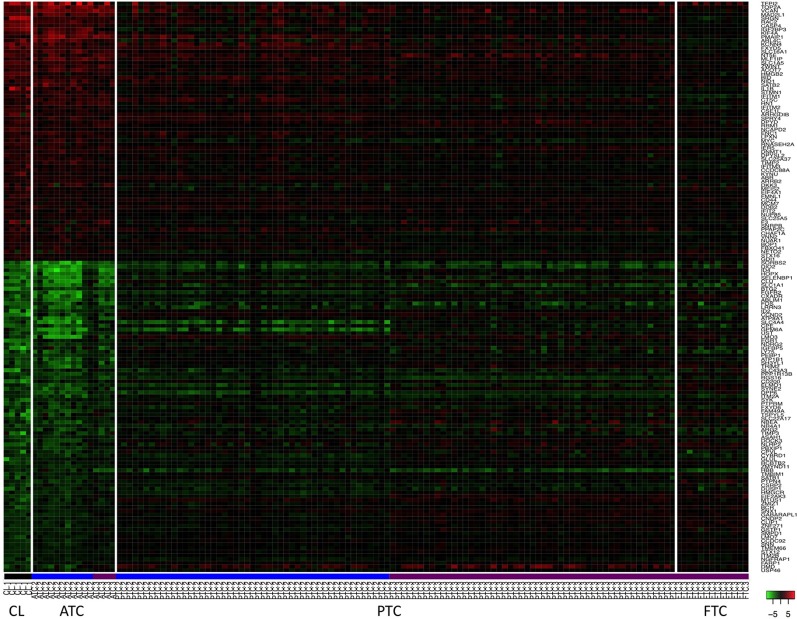
**Heatmap of 145 mRNA expressions in thyroid cancer cell lines (CL) and in human thyroid tumors**. These genes were the most commonly up and down-regulated genes in ATC and in the cell lines. The level of modulation of these genes in PTC and FTC are also shown. Profiles of the five cell lines are underlined in black. Profiles of the samples hybridized on Affymetrix HG U133 Plus 2.0 platform and on Affymetrix HG U133A platform are underlined in blue and violet respectively. Genes are indicated by their official gene symbols.

To further investigate the similarity between ATC and cell lines, their mRNA expression profiles, obtained on different microarray platforms, were compared. Rank Products and SAM were used to find genes significantly regulated in the same direction or in the opposite direction comparing ATC with cell lines. Sixty-one genes were found to be up-regulated in both ATC and cell lines and eighty-four genes were found to be commonly down-regulated (Figure 3). Eighteen genes were regulated in an opposite direction between ATC and cell lines.

To gain more insight into those regulations, a DAVID pathway analysis was performed (Supplementary information B). Some common up-regulated genes in ATC and cell lines are involved in DNA replication and DNA metabolic processes, e.g., *TOP2A, MCM7, CHAF1A*, or *HMGB2*. Most of the 18 genes that were regulated in an opposite direction were down-regulated in cell lines but up-regulated in ATC. They are involved in MHC class II pathway or in cell adhesion, e.g., *HLA-DPB1, HLA-DQB1, HLA-DRA, ICAM1, CCL2*, or *COL6A3.*

## Discussion

In this work, the aim was to further investigate the characteristics for different human thyroid cancer cell lines. First, genetic alterations in these cell lines were confirmed. Second, regulated pathways in these cell lines were further explored. Third, correlations, based on gene expressions, between *in vitro* human thyroid cell lines and *in vivo* human thyroid tumors were analysed. Finally, the similarity between cell lines and ATC was further explored on the gene level.

### Mutational status

We have investigated the mutational status of 10 commonly mutated genes in thyroid cancer by using six different cell lines. STR analyses on these cell lines (van Staveren et al., 2007 and Supplementary information C) showed that they were identical to those published by Schweppe and coauthors (Schweppe et al., 2008). No differences with reported literature data were observed for the mutational status of these genes in BCPAP, FTC133, TPC1, and 8505C cell lines. However, a difference could be observed for the status of BRAF in WRO and *PI3KCA* in K1 cells.

For WRO cells described as a FTC cell line, the *BRAF* T1799A (V600E) mutation was detected although these cells were originally described as *BRAF* wild-type (Namba et al., 2003; Xu et al., 2003) and are still frequently used as such today (Palona et al., 2006; Liu et al., 2007; Burrows et al., 2010; Hou et al., 2010). The presence of a *BRAF* mutation in these cells was nevertheless reported by Schweppe and coworkers (Schweppe et al., 2008) and they demonstrated that the cell line was not contaminated by another cell line harboring this mutation. It has been suggested that two different WRO cell lines were distributed. The WRO cells from Schweppe's laboratory has the same DNA profile (STR analysis) than ours (van Staveren et al., 2007; Schweppe et al., 2008), but is different from that of the European Collection for Cell Culture. The presence of this mutation in WRO cells was unexpected because this cell line has been derived from a FTC, and the V600E *BRAF* mutation is described as a specific characteristic for PTC. This might suggest that, in spite of its initial diagnosis of follicular cancer, the tumor at the origin of the creation of the WRO cell line would have rather been a papillary type. The presence of a mixture of *BRAF* mutated and non-mutated cells in the original tumor sample cannot be excluded. However, it may also be proposed that this cell line acquired this mutation *in vitro*, during its successive divisions in an artificial environment of selection (van Staveren et al., 2007, 2009). These observations question the representativity and the utility of this cell line as a model for human FTC and show the necessity of a systematic check of the cell lines. Furthermore, the V600E *BRAF* mutation is not specific of thyroid cancer. The origin of the WRO cell line as a thyroid cancer cell line is not completely certified.

For the K1 cell line, a mutation in the *PI3KCA* gene has been reported previously. By sequencing, the presence of a G1624A substitution in codon 542 (E542K) was confirmed in accordance with previously published data (Garcia-Rostan et al., 2005; Meireles et al., 2007). In another publication, Ricarte-Filho et al. (Ricarte-Filho et al., 2009) reported the presence of a E545K (G1633A) mutation in these cells. We could observe a glutamic acid at position 545. Thus, the G1633A mutation might potentially exist but was never described as a new mutation by Ricarte–Filho et al.

### Pathway analysis of the cell lines

Pathway analysis of mRNA expression profiles in WRO, FTC133, BCPAP, TPC1, and 8505C cell lines was performed. In general, the cell lines abrogate gene expressions which are not necessary for their propagation in *in vitro* cultures and express genes that promote in growth and survival. The common loss of MHC2 biosynthesis and, in some cell lines, of the immune response, can be explained because they would be not necessary for a cell in culture. The common downregulation of the negative regulation of the cell death/apoptosis pathway might be explained by an ineffective feedback resulting from an excess of proliferation.

Except for FTC133 cells, a common overexpression of ribosomal biosynthesis and translation pathways were observed. This may reflect the increase in proliferation of the cancer cells induced by gene mutations. However, some recent studies suggest that the up-regulation of the ribosomal biosynthesis may induce cancer by a downregulation of the cell tumor suppressor potential (Montanaro et al., 2012). For our data, the two explanations are possible. However, the growth rate of the FTC133 cell line is higher than for the other cell lines [Dom et al. (submitted)]. For this cell line, an overexpression of the global RNA processing including alternative splicing was observed. This might allow the strong increase in proliferation of this cell line.

### Correlations between mRNA expression profiles of *in vitro* thyroid cancer cell lines and mRNA expression profiles of *in vivo* thyroid tumors

The correlations of the mRNA expression profiles between cell lines and ATC were significantly higher than those compared to other *in vivo* differentiated thyroid tumors (PTC, FTC). By using data derived from different microarray platforms and from two different laboratories, thereby including a larger number of *in vivo* tumors, the current data confirm our previous finding that mRNA expression profiles from human thyroid tumor cell lines with different origins are the closest to undifferentiated *in vivo* tumors (van Staveren et al., 2007). This is explained by an *in vitro* evolution of the cell lines that should be taken into account when extrapolating results obtained from these cells. An *in vitro* cell line is the result of the selection of the most rapidly proliferating and resistant cells during numerous passages. Cell lines contain one cell type, whereas *in vivo* tumors are complex mixtures of various cell types. Therefore, it might be argued that the comparisons between thyroid cancer cell lines and *in vivo* thyroid tumors could be affected by the presence of different cell types such as for instance lymphocytes, endothelial cells. However, in this study only genes were compared that were expressed in thyrocytes, i.e., were expressed in both thyroid cancer cell lines and in primary thyrocytes. This should reduce the impact of the potential presence of different cell types but, unfortunately, it cannot assure the complete elimination of a background due to commonly expressed genes in the different cell types.

### Analysis of the genes commonly and oppositely modulated in ATC *in vivo* and cancer cell lines

Genes commonly up-regulated in ATC and cell lines were involved in DNA replication and DNA metabolic processes. The correlation, based on mRNA expression profiles, between cell lines and ATC thus seems to be due to the strong proliferation capacity of these two types of cells. Indeed, ATC is one of the most proliferating human tumors and human thyroid cancer cell lines have a strong proliferation capacity. In our data this property was linked to ribosomal biosynthesis and translation pathways for four cell lines. As expected, the genes found regulated in an opposite direction between ATC and cell lines might reflect the differences between *in vivo* tumors and *in vitro* cultures. They are involved in the MHC class II pathway or in cell adhesion. As mentioned above in the pathway analysis of the cell lines, they abrogate gene expressions which are not necessary for their survival and growth in *in vitro* cultures. The increase of those genes in ATC, and the implication of this increase to growth and survival of the tumor *in vivo*, would be interesting to explore. However, there are a number of factors which could also explain this increase. For instance, the potential presence of immune cells, which express MHC class II in the tumor or the activation of the MHC class II pathway in these cells by an inflammatory process, might explain an increase in these genes in ATC. In line with this, thyrocytes indeed can express MHC class II after an inflammatory stimulation and inflammation is often associated with tumor development. Furthermore, the increase of expression might also be due to a combination of the factors.

## Conclusions

In this work, the aim was to further investigate the characteristics of human thyroid cancer cell lines derived from different origins. First, genetic alterations in six cell lines were in line with literature data and furthermore, the BRAF mutation in WRO cells questions this cell line as a model for FTC, indeed this mutation does not occur in FTC. Second, in five cell lines the MHC class II pathway was down-regulated and in four of them, ribosome biosynthesis and translation pathways were up-regulated. Third, by using microarray data from ATC, PTC, and FTC, we have preliminary found that mRNA expression profiles of human thyroid cancer cell lines, originally derived from ATC, poorly differentiated PTC, FTC, or PTC, were closer to ATC. Specifically, we found genes commonly up-regulated in ATC involved in DNA replication which were also up-regulated in the thyroid cancer cell lines, in accordance with their higher proliferation rate *in vitro*. In summary, these results would suggest that some human PTC or FTC derived cell lines (i.e., TPC-1) might have partially lost their original DNA synthesis/replication regulation mechanisms during their *in vitro* cell adaptation/evolution. Also, further comparisons using microarray data set from poorly differentiated thyroid cancers are needed and could be a near future goal in our laboratory.

During our study, the origin of the WRO cell line as thyroid cancer cell line has been questioned. However, the pathway analysis and the comparisons of expression profiles showed similar results for WRO cell line and the other thyroid cancer cell lines. To address the origin of the WRO cell line, more complete analyses (including cell lines from different cancer types) could be a near future goal in our laboratory.

### Conflict of interest statement

The authors declare that the research was conducted in the absence of any commercial or financial relationships that could be construed as a potential conflict of interest.

## Supplementary material

### Supplementary information A

**DAVID pathway analysis of the genes modulated in each human thyroid cancer cell line**. Up, analysis of pathways using all the upregulated genes (>0.58 log_2_) in the cell line. Down: analysis of pathways using all the downregulated genes (<−0.58 log_2_) in the cell line.

### Supplementary information B

**DAVID pathway analysis of the genes commonly and oppositely modulated in human thyroid cancer cell lines and ATC**. Up: analysis of pathways using the 61 up-regulated genes that were upregulated in both. Down: analysis of pathways using the 84 genes down-regulated in both ATC and cell lines. Modulation: analysis of pathways using the 18 genes that were regulated in an opposite direction comparing cell lines with ATC.

### Supplementary information C

**DNA profiling by STR analysis of the K1 cell line**.
